# Editorial: Cross Adaptation and Cross Tolerance in Human Health and Disease

**DOI:** 10.3389/fphys.2018.01827

**Published:** 2019-01-08

**Authors:** Ben James Lee, Oliver R. Gibson, Charles Douglas Thake, Mike Tipton, John A. Hawley, James David Cotter

**Affiliations:** ^1^Occupational Performance Research Group, Institute of Sport, University of Chichester, Chichester, United Kingdom; ^2^Department of Life Sciences, Centre for Human Performance, Exercise and Rehabilitation, Brunel University London, Uxbridge, United Kingdom; ^3^Faculty of Health and Life Sciences, Coventry University, Coventry, United Kingdom; ^4^Extreme Environments Laboratory, Department of Sport and Exercise Science, University of Portsmouth, Portsmouth, United Kingdom; ^5^Mary MacKillop Institute for Health Research, Australian Catholic University, Melbourne, VIC, Australia; ^6^School of Physical Education, Sport and Exercise Sciences, University of Otago, Otago, New Zealand

**Keywords:** heat, adaptation, preconditining, hypoxia, nutrition, dehydration

Human physiological responses to heat, cold, hypoxia, microgravity, hyperbaria, hypobaria, and fasting are well-studied in isolation. However, in the natural world these stressors are often combined or experienced sequentially (Tipton, 2012). Studies examining human responses to these more realistic, yet relatively complex, circumstances remain sparse, but could provide important insights into an emerging area within human physiology: cross-adaptation (Figure 1) (Lunt et al., 2010; Gibson et al., 2017). Much of the current state of knowledge involves data demonstrating benefits of exercising in hot conditions, prior to performance in hypoxia (Heled et al., 2012; Lee et al., 2014a,b, 2016; Gibson et al., 2015; White et al., 2016; Salgado et al., 2017), with cold to hypoxia (Lunt et al., 2010), hypoxia to heat (Sotiridis et al., 2018), combined stressors (Takeno et al., 2001; Neal et al., 2017), and more mechanistic (signaling) data from animal models exposed to substantive volumes of stress (Maloyan and Horowitz, 2002, 2005). The role of nutrient availability and the nutrient-exercise interactions which drive phenotypic adaptations to skeletal muscle exposed to a multitude of stressors is also a growing field of interest (Hawley et al., 2018). This research topic includes publications which address both clinical and exercise-centric aspects allied to Cross-adaptation and Cross-tolerance in Human Health and Disease.

**Figure 1:**
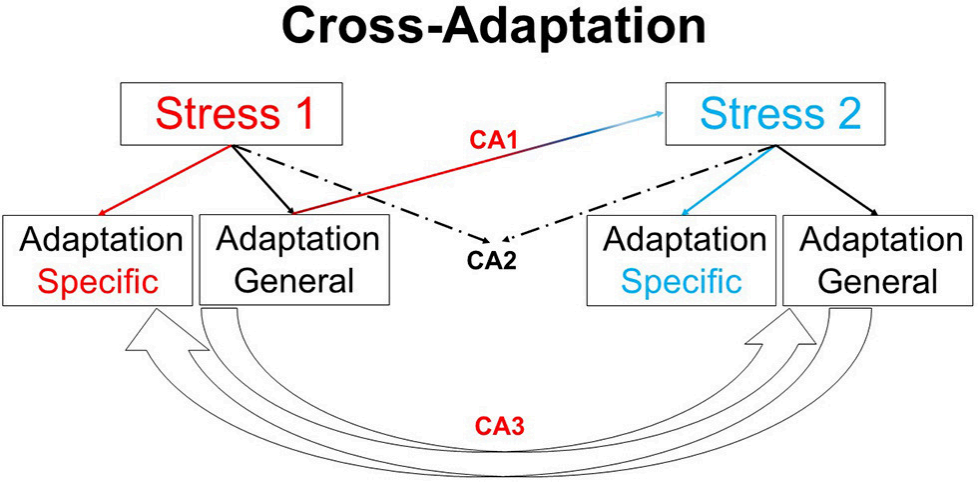
Overview of cross-adaptation. Adaptation to one stimulus provides cross tolerance to another (CA1). For example, heat acclimation enhances tolerance to hypoxia, and may enhance exercise performance under temperate conditions. CA2 highlights the combined adaptive effects of two stimuli in providing beneficial responses to a third variable, e.g., heat acclimation alongside exercise training may enhance left ventricular function. The potential for adaptation to one stressor enhancing adaptation to other stressors (CA3), such as the manipulation of nutrient availability to enhance or amplify skeletal muscle responses to training, remains an interesting area for future work. Finally, the use of one stimulus to model the responses to the same or similar stimulus in another circumstance (e.g., high altitude hypoxia as model to examine physiological responses in an intensive care unit) follows the same conceptual premise as cross adaptation.

An excellent primer covering aspects of preconditioning, short and long term heat acclimation cross-tolerance, and heat acclimation memory in both animal and human experimental models has been provided by Professor Horowitz of the Hebrew University. This extensive review of the cellular and molecular responses underpinning cross-adaptation concludes that the dynamic epigenetic phenomenon not only induces long-lasting cross-tolerance, but enables preservation of its physiological beneficial features in a dormant manner. This conclusion is based on an increasing understanding of epigenetic mechanisms of cross-adaptation and the notion of Heat Acclimation-Mediated Cross-Tolerance vs. Preconditioning-Induced Cross-Tolerance, with a specific focus on the extensive publications from the Horowitz laboratory providing evidence for the role of hypoxia inducible 1-alpha (HIF-1α) and heat shock proteins (HSPs) in cytoprotection in the ischemic heart. In short, a rapid, short acclimation stimulus re-establishes the physiological, protected heat acclimation phenotype. The transcriptional machinery, however, is a continuum and as such our knowledge of the epigenetic within-life dynamic mechanisms involved, and their rates of both development and decay, is still in its infancy.

The review by Horowitz sets up three original investigations with a clinical focus. Pollak et al. provide original experimental data that evidences the protective effect of heat acclimatization combined with modest exercise training, performed in “free living” conditions, against diastolic dysfunction imposed by ischemic/reperfusion insults in humans. The surgical theme is continued by Barrington et al., who demonstrate the efficacy of using orthopedic perioperative hypoxic air inhalation to prime the local HSP system and attenuate reperfusion stress in a manner similar to that of the current ischemic preconditioning intervention used prior to knee replacement surgery. The benefit of hypoxic air inhalation being that it comes without negative post-surgical side-effects, e.g., delayed wound healing, vascular injury, muscular damage, and greater post-operative pain that can arise as a consequence of ischemia. Hypoxic air inhalation was also the subject of investigation for Morrison et al.; they identify that during 21 days of bed rest, sleep macrostructure is negatively affected, the apnoea-hypopnea index increases, and breathing stability worsens. Each of these metrics become independently exacerbated by continuous exposure to hypoxia. Given the importance of sleep for many clinical and exercise applications where cross-adaptation/tolerance is investigated, interventions using chronic stress such as prolonged hypoxia should be mindful of the potential confounding/maladaptive issues arising from its use.

Exercise related cross-adaptation is studied most extensively within this Research Topic. Leckey et al. present interesting experimental work describing the effects of a 1,3-butanediol acetoacetate (ketone) diester on performance in professional cyclists. In comparison to a placebo, pre-exercise ingestion of the diester results in an impairment in time trial performance that is associated with gut discomfort and higher perception of effort concurrent with elevated serum β-hydroxybutyrate, serum acetoacetate and urine ketone concentrations. Continuing the exercise and nutrition cross-adaptive theme, papers by Akerman et al. and Neal et al., investigate the role of fluid balance, and more specifically dehydration as an additional stressor during exercise-heat stress. To understand mechanisms by which manipulating fluid balance may augment adaptation to exercise-heat stress, Akerman et al. prescribed calisthenics in temperate conditions, in hot conditions whilst euhydrated, and in hot conditions with dehydration. Following the three conditions the authors conclude that transient dehydration with heat potentiates short-term (24-h) hematological (hypervolemic) and cardiovascular (hypotensive) outcomes. Neal et al.'s work extends understanding of the relevance of fluid intake during heat acclimation protocols (repeated exercise heat stress) by identifying that when thermal-strain is matched, permissive dehydration which induces a mild, transient, hypohydration does not reliably affect the acquisition or decay of heat acclimation, or endurance performance parameters. Consequently, the current guidance relating to inducing heat adaptation using acclimation remains salient i.e., irrespective of hydration status, trained individuals require >5 days to optimize heat acclimation.

Since the emergence of seminal work in the field, heat has been the most widely considered stressor for cross-adaptation. Lee and Thake follow a previous publication in the area (Lee et al., 2016) with important insights into the responses of circulating inflammatory markers during acclimation to control, hypoxic or hot conditions, and subsequent hypoxic stress tests. Whilst heat acclimation induces more immediate and greater changes in monocyte HSP72 in comparison to equivalent training in hypoxia, neither regime attenuated the systemic cytokine response or intestinal damage following acute exercise in hypoxia. This experiment identified that 10 days of fixed-work acclimation does not induce full cytoprotective adaptation, and cellular acclimation homeostasis had yet to be achieved. Further studies are therefore warranted to determine the optimal heat “dose” to maximize the constitutive HSP72 reserves and achieve the potent cross-tolerance effects observed in rodent models (Maloyan and Horowitz, 2002, 2005). Once the optimal dose and associated rates of development and decay are better understood in humans, cross-adaptive interventions aimed at enhancing hypoxic tolerance and operational effectiveness in human occupational, military, and sporting scenarios can be explored. These applied fields can draw upon the data of Tuttle et al. who demonstrate that an acute bout of hot downhill running is an effective preconditioning strategy that ameliorates physiological strain, soreness, and Hsp72 and Hsp90α mRNA responses of muscle and leukocytes, to a subsequent bout of hot downhill running — these responses being indicative of cross-adaptation. The ability to elicit physiological and cellular protection to exercise heat stress following just a single bout of exercise is appealing for those individuals where extensive periods of time prior to exercise in extreme environments is not possible. For example, cross-adaptation and preconditioning principles could be considered when examining interventions aimed at preparing vulnerable populations, such as the elderly and those with chronic non-communicable diseases, before the onset of debilitating heatwave events that increase morbidity and mortality in these populations (Kenny et al., 2010). For the researcher, these data also valuably demonstrate the efficacy of utilizing circulating tissue (leukocytes) in place of muscle samples to infer changes in the heat shock response.

The final inclusion in this research topic brings the collection full circle by considering exercise and clinical cross overs. Sahl et al. investigate how excessive repeated prolonged exercise influence low-grade inflammation and adipose tissue anti-inflammatory macrophage content in six older male recreationally-trained cyclists. The data support the conclusion that regular prolonged exercise does not influence abdominal adipose tissue inflammation, but a higher plasma IL-6 concentration concurrent with a trend toward higher insulin resistance and decreased VO_2peak_, implies that excessive exercise probably attenuates its potential anti-inflammatory effects. The balance between successfully inducing an adaptation and providing too great a stimulus and thereby mitigating the desired response is a delicate balance, and all research into cross-adaptation should be mindful of the challenges of prescribing appropriate exercise intensities, particularly when dealing with individuals of widely varying levels of fitness and when seeking to complement exercise with environmental stress such as heat and hypoxia, or nutritional and dehydration manipulations.

These articles add to our understanding of the whole body, systemic and molecular responses to a multitude of stressors that interact with one another under the cross-adaptation paradigm. The sporting, occupational, and clinical relevance of these areas is only recently being discovered and we look forward to new applied and mechanistic data in this growing area.

## Author Contributions

BL and OG: Design and scripture. CT, MT, JH and JC: Completion and correction.

### Conflict of Interest Statement

The authors declare that the research was conducted in the absence of any commercial or financial relationships that could be construed as a potential conflict of interest.
